# Effectiveness of Sinovac vaccine against SARS-CoV-2 (CoronaVac) in reducing in-hospital mortality in individuals with COVID-19 and schizophrenia: a retrospective cohort study

**DOI:** 10.1186/s12889-025-25979-w

**Published:** 2025-12-17

**Authors:** Yuyang Gao, Char Leung

**Affiliations:** 1 School of Biological Sciences, University of Leicester, Leicester, UK; 2 Division of Public Health and Epidemiology, School of Medical Sciences, University of Leicester, George Davies Centre, 15 Lancaster Road, Leicester, LE1 7HA UK

**Keywords:** COVID-19, Schizophrenia, Vaccine, Mortality, Sinovac

## Abstract

**Background:**

COVID-19 causes a huge burden on global health and is associated with mental health issues. Schizophrenia patients have impaired immune system, which leads to weaker resistance to virus. The effectiveness of CoronaVac in reducing in-hospital mortality in individuals with COVID-19 and schizophrenia is not well understood. The aim of this study was to determine the effects of Sinovac vaccine on patients with COVID-19 and schizophrenia, to guide clinicians and policies.

**Methods:**

This retrospective cohort study used patients’ data selected from the database Sistema de Informação da Vigilância Epidemiológica da Gripe (SIVEP-Gripe). Demographic and clinical conditions data were collected as covariate data. To compare intergroup descriptive statistics, Student *t*-test, Mann-Whitney test, and Fisher’s exact test were respectively used. Multivariable logistic regression was done for the primary outcome of in-hospital all-cause mortality, and adjusted odds ratio (aOR) was calculated. Hosmer–Lemeshow test, Receiver Operating Characteristic analysis, multivariable collinearity analysis, Akaike information criterion, and c-statistics were used to evaluate models.

**Results:**

We report findings for eligible 1,983 patients, from SIVEP-Gripe, received Sinovac vaccines or no vaccines at all. Sinovac vaccination lowered in-hospital mortality by 52.0% in these patients (aOR = 0.480, 95%CI 0.349–0.660, *p* < 0.001). Moreover, each year of age increased mortality risk (aOR = 1.029, 95% CI 1.022–1.036). Patients in Northeast and North Brazil faced higher mortality (aOR = 1.744 and 2.811, respectively) compared to the Southeast. Immunocompromised status (aOR = 3.512, 95%CI 1.516–8.134, *p* = 0.003) and neurologic disease (aOR = 1.659, 95%CI 1.291–2.133, *p* < 0.001) independently elevated mortality risk.

**Conclusion:**

Sinovac vaccine can significantly reduce the in-hospital mortality in individuals with SARS-CoV-2 infection and schizophrenia, compared to those not-vaccinated.

## Introduction

 COVID-19, or SARS-CoV-2, is a highly infectious and dangerous coronavirus that first appeared in late December 2019, which triggered a global outbreak of acute respiratory illness [1]. COVID-19 is an enveloped, single stranded, RNA virus [2]. The S glycoprotein on the surface of coronavirus is considered crucial to bind ACE2 receptors, which are abundant in human lower respiratory tract [3]. COVID-19 patients release virus-laden droplets when speaking, while smaller particles known as aerosol remain suspended in the air for extended periods, which will be inhaled by others, penetrating deep into the lungs [4]. The pathological changes in COVID-19 patients happen mainly in lungs, including tracheobronchitis characterized by mononuclear inflammation, bilateral diffuse alveolar damage, submucosa congestion, presence of desquamated pneumocytes and hyaline membranes, fibrin deposits [5]. Based on virology characteristics and photomicroscopic changes caused by COVID-19, patients usually present fever, cough, shortness of breath [6]. Less common symptoms include haemoptysis, confusion, headache, muscle ache, nausea and vomiting [7].

Schizophrenia, a complex mental health disorder imposing a substantial public health burden, has seen its diagnostic criteria evolve across various editions of the International Classification of Diseases (ICD) [8]. In ICD-11, the diagnostic criteria for Schizophrenia is similar, which includes several aspects: Core symptoms, additional symptoms, duration criterion, and so on [8]. Studies indicate that schizophrenia impact more than just mental health; patients diagnosed with the condition have a lifespan 12–15 years shorter than the general population, which is a greater loss than most cancers [9].

Some studies showed that schizophrenia is a risk factor for COVID-19 [10, 11]. An American nationwide database analysis including 61 million individuals showed that patients recently diagnosed with schizophrenia had a significantly higher susceptibility for COVID-19 (adjusted odds ratio 7.34, *P* < 0.001) [11]. In fact, the impacts can be mutual between schizophrenia and COVID-19. Additionally, a mendelian randomization analysis suggests the possibility that severe COVID-19 can increase the risk of schizophrenia, but does not support the causality between the two [12]. That is to say severe COVID-19 is associated with increased schizophrenia risk, but does not necessarily cause schizophrenia. The mechanism is complex, but there is evidence showing that multiple immune system changes and central neural system lesions induced by SARS-CoV-2 infection may potentially cause psychosis, including schizophrenia. One recent study summarized that coronavirus invades central neural system, affecting mental health by various mechanisms, including hyperinflammation, CNS infection and penetration, and the psychosocial stress [13]. In reality, the COVID-19 pandemic are also associated with sub-syndromal mental health issues, due to varying reasons including isolation, societal disruption, and so on [14, 15].

Sinovac vaccine is an inactivated SARS-CoV-2 vaccine (CoronaVac), developed and produced by Sinovac Biotech Ltd. According to Phase 1–2 trials conducted in China, Sinovac demonstrated safety and immunogenicity in participants aged 18–59, with observable immune responses emerging 14 days after the second dose [16]. Brazil launched its Covid-19 vaccination campaign on January 18, 2021, initially utilizing the CoronaVac-Sinovac/Butantan vaccine and the ChAdOx-1 vaccine [17]. 74.8% overall Brazilians had been fully vaccinated for SARS-CoV-2, by 1 st March 2022 [17].

Influenza Epidemiological Surveillance Information System (SIVEP-Gripe), which provides open access to anonymized data, is used in Brazil for COVID-19. In fact, this system has been established by Brazilian government since the 2009 Influenza A (H1N1) pandemic. Patients admitted to public or private hospitals with a reportable disease are registered in SIVEP-Gripe, where basic demographic and medical information is systematically recorded using a standardized form designed for severe respiratory disease hospitalizations [18].

## Objectives

To assess the effectiveness of the Sinovac vaccine in lowering in-hospital mortality among schizophrenia patients with COVID-19, we performed a retrospective cohort analysis using data from Brazil’s nationwide SIVEP-Gripe registry. The findings could inform clinical decisions on adjusting therapeutic approaches for this vulnerable population and emphasize the importance of vaccination to mitigate mortality risks in COVID-19-infected individuals with schizophrenia.

## Method

### Study cohort

The study population were selected from the database SIVEP-Gripe, https://opendatasus.saude.gov.br. The inclusion criteria include: (a) PCR confirmed positive for SARS-CoV-2, (b) Diagnosed as schizophrenia, (c) Patients either discharged from hospital or died, (d) Received Sinovac vaccine or no vaccine against SARS-CoV-2. The exclusion criteria include: (a) Received other vaccines against SARS-CoV-2, (b) missing date of admission or death/discharge.

### Grouping and outcome

The exposure group refers to patients vaccinated with Sinovac vaccine; The control group refers to patients not vaccinated with any vaccine against SARS-CoV-2. The primary outcome was in-hospital all-cause mortality, measured by odds ratio (OR). Outcome measures were adjusted for baseline statistics, such as demographic features, and underlying conditions.

### Definition of measurements and data handling

Demographic and clinical conditions data were collected as covariate data from SIVEP-Gripe. These data included: age, gender, ethnicity, region, signs and symptoms (including fever, cough, sore throat, dyspnea, low oxygen saturation, respiratory discomfort, diarrhea, vomiting, abdominal pain, anosmia, and ageusia), underlying conditions (including cardiovascular disease, asthma, immunocompromised, chronic liver disease, chronic neurologic disease, chronic renal disease, diabetes, and obesity), usage of mechanical ventilation and anti-virus drugs, admission to ICU or not, vaccinated or not. Patients were categorized as having schizophrenia based on the SIVEP-Gripe database, where clinical providers report schizophrenia.

Patients’ ages are calculated as the difference between date of admission and date of birth. The sex and ethnicity were self-identified by patients. The ethnicity contains 6 types, including African, Asian, Caucasian, indigenous, Mixed (Hispanic), and NA (data missing). Mixed (Hispanic) were chosen as the reference for ethnicity because this group constitutes the largest ethnic population in Brazil. The region contains 5 areas: Southeast, South, Center West, Northeast and North. Southeast region was selected as the reference for region due to its generally greater accessibility to healthcare.

For missing data on clinical characteristics, such as signs and symptoms, we assumed the absence of the clinical condition, which was consistent with the methodology used in a prior study analyzing the same database [19]. For missing data on categorical variables, a new variable called “missing” was created. For missing data on variables “Age”, which is continuous, we also created a new variable “Age missing”.

### Statistical analysis

To summarize descriptive statistics in vaccination group and non-vaccination group, we used mean ± standard deviation (SD) for normally distributed continuous variables, median with interquartile range (IQR) for non-normally distributed continuous variables or ordinal variables, and proportion for categorical variables. To compare descriptive statistics in the two groups, we used Student *t*-test for continuous variables, Mann-Whitney test for ordinal variables or non-normally distributed continuous variables, and Fisher’s exact test for categorical or dichotomous variables. For the outcome, we used a multivariable logistic regression model to calculate the OR for in-hospital mortality. The goal of our model building is to evaluate the association between Sinovac and mortality while adjusting for confounders.

For covariate selection, each covariate was individually analyzed in a univariable logistic regression model with “in-hospital death” as the outcome, yielding crude odds ratios (ORs). Covariates with a p-value < 0.1 were then selected for inclusion in a multivariable logistic regression model, where they were analyzed together to produce adjusted ORs. Signs and symptoms, ICU admission, and ventilation usage were excluded from the regression analysis as they are mediators rather than confounders. We used the Hosmer–Lemeshow test to evaluate the goodness-of-fit for the logistic regression model, and the Area under the receiver operating characteristic curve (ROC) to evaluate the discrimination. Collinearity of the logistic regression model was tested using variance inflation factor (VIF). *P* < 0.05 was considered to be statistically significant. VIF < 10 was considered to be no severe multicollinearity. We also calculated the Akaike information criterion (AIC), c-statistics values to better describe the model as well. Finally, we conducted the sensitivity analysis, redoing our analysis by focusing on those subjects who are in the southeast region, because of the convenience of accessing medical resources in this region.

All calculations were performed on SPSS version 29.0.2.0, GraphPad Prism version 10.3.1 and R 4.3.1. No ethics approval is required due to the de-identified, public data gathered from SIVEP-Gripe.

## Results

**Fig. 1:**
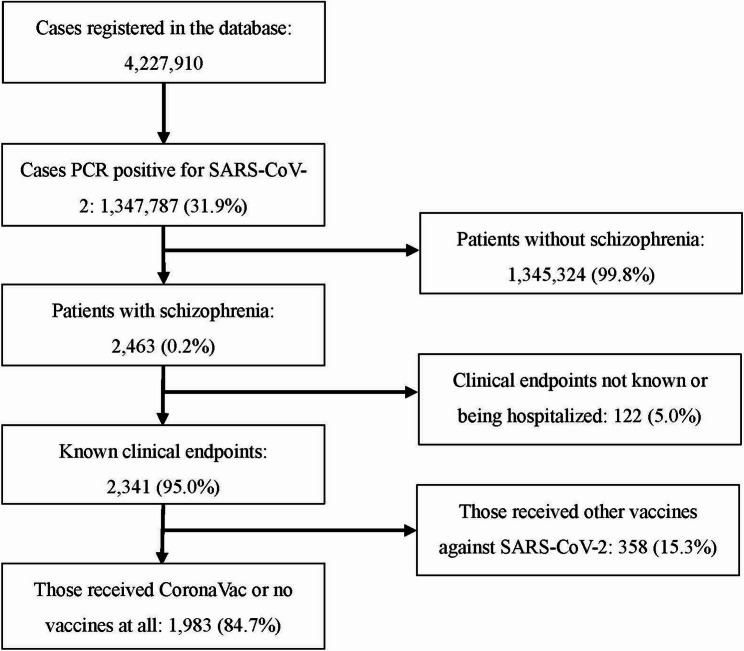
Flowchart of case inclusion. Data was extracted from SICEP-Gripe database and was publicly available

SIVEP-Gripe system recorded a total of 4,227,910 cases, with 1,347,787 (31.9%) testing positive for SARS-CoV-2 via PCR. All COVID-19 cases were recorded since 2020, and were gathered on December 26th, 2024. Among these 1,347,787 patients, 2,463 were reported as schizophrenia (0.2%), 1,345,324 were not reported as schizophrenia (99.8%). Among 2,463 patients, 122 had unclear clinical endpoints or were still hospitalized during the period of study (5.0%). 2341 patients had known clinical endpoints: dead or discharged (95.0%). Among those 2341 patients, 358 received other vaccines against SARS-CoV-2, rather than Sinovac vaccines (15.3%), 1,983 patients received Sinovac vaccines or no vaccines at all (84.7%). Eventually, this study included 1,983 patients that reached all selection criteria (Fig. 1).

Descriptive statistics of patients were summarized in Table 1. Generally, 207 (10.4%) cases were vaccinated by Sinovac, 1776 cases (89.6%) were not vaccinated by Sinovac. For demographic data, two groups were well balanced for sex (*p* = 0.4606). No significant differences were found between two groups in Age missing (*p* > 0.9999), region for southeast (*p* = 0.9146), center west (*p* = 0.5358), northeast (*p* = 0.0530), north (*p* = 0.1700), and ethnicity for indigenous (*p* > 0.9999), African (*p* = 0.3148), Asian (*p* = 0.7134), missing (*p* = 0.7009). There is significant difference in in-hospital mortality rate between vaccinated group (38.2%) and not-vaccinated group (47.1%) (*p* = 0.0151). For signs and symptoms, vaccinated patients had less onset than not-vaccinated patients, including fever (49.3% vs. 58.6%, *p* = 0.0115), and dyspnea (58.0% vs. 72.8%, *p* < 0.0001). For underlying conditions, two groups were generally balanced, with no significant differences in asthma (*p* > 0.9999), Immunocompromised (*p* = 0.2329), chronic liver disease (*p* = 0.7479), chronic neurologic disease (*p* = 0.1128), chronic renal disease (*p* = 0.3890), and diabetes (*p* = 0.3894). Not surprisingly, not-vaccinated patients were more likely to use antiviral drugs than vaccinated patients (antiviral drugs use rate 10.5% vs. 1.4%, *p* < 0.0001). No significant differences were noticed between the two groups in ICU admission (*p* = 0.1045), use of invasive ventilation (*p* = 0.0635), and use of non-invasive ventilation (*p* = 0.2697).

**Table 1:** Summary of the characteristics of patients in the cohort

**Characteristics**	No. participants		
	Vaccinated, *n* = 207	Not vaccinated, *n* = 1776	p value
Died, no. (%)	79 (38.2%)	836 (47.1%)	0.0151
Demographic			
Median age, y (IQR)	71.8 (65.2–77.1)	58.6 (47.6–67.8)	< 0.001
Age missing, no.	1	1	> 0.9999
Sex, %			
Male	111 (53.6)	1000 (56.3)	0.4606
Female	96 (46.4)	776 (43.7)	
Region, no. (%)			
Southeast	101 (48.8)	861 (48.5)	0.9146
South	62 (30.0)	382 (21.5)	0.0080
Center West	17 (8.2)	177 (10.0)	0.5358
Northeast	26 (12.6)	322 (18.1)	0.0530
North	1 (0.5)	34 (1.9)	0.1700
Ethnicity, no. (%)	*n* = 168	*n* = 1462	
Indigenous	0 (0)	1 (0.07)	> 0.9999
African	14 (8.3)	90 (6.2)	0.3148
Asian	1 (0.6)	19 (1.3)	0.7134
Caucasian	114 (67.9)	797 (54.5)	0.0010
Mixed	39 (23.2)	555 (38.0)	0.0001
Missing	39 (18.8), *n* = 207	314 (17.7), *n* = 1776	0.7009
Signs and symptoms, no. (%)			
Fever	102 (49.3)	1040 (58.6)	0.0115
Cough	132 (63.8)	1167 (65.7)	0.5891
Sore throat	22 (10.6)	226 (12.7)	0.4379
Dyspnea	120 (58.0)	1293 (72.8)	< 0.0001
Low SpO2 (< 95%)	134 (64.7)	1227 (69.1)	0.2059
Respiratory discomfort	104 (50.2)	969 (54.6)	0.2394
Diarrhea	22 (10.6)	180 (10.1)	0.8083
Vomit	10 (4.8)	111 (6.3)	0.5386
Abdominal pain	9 (4.3)	65 (3.7)	0.5632
Fatigue	48 (23.2)	319 (18.0)	0.0724
Anosmia	6 (2.9)	71 (4.0)	0.5687
Ageusia	7 (3.4)	81 (4.6)	0.5914
Underlying conditions, no. (%)			
Chronic cardiovascular disease	78 (37.7)	478 (26.9)	0.0014
Asthma	4 (1.9)	39 (2.2)	> 0.9999
Immunocompromised	5 (2.4)	25 (1.4)	0.2329
Chronic liver disease	3 (1.4)	23 (1.3)	0.7479
Chronic neurologic disease	42 (20.3)	283 (15.9)	0.1128
Chronic renal disease	8 (3.9)	51 (2.9)	0.3890
Diabetes	44 (21.3)	431 (24.3)	0.3894
Obesity (BMI > 30)	7 (3.4)	194 (10.9)	0.0002
Treatment and therapeutic (%)			
Use of antivirals	3 (1.4)	187 (10.5)	< 0.0001
Admission to ICU	81 (39.1)	801 (45.1)	0.1045
Invasive ventilation	49 (23.7)	532 (30.0)	0.0635
Non-invasive ventilation	104 (50.2)	818 (46.1)	0.2697

In the step of filtering interested variables for multivariable logistic regression model, 9 variables were selected, which were: Age (crude OR [cOR] 1.026, 95%CI 1.020–1.032), northeast region (cOR 1.425, 95%CI 1.114–1.823), north region (cOR 2.463, 95%CI 1.212–5.007), NA ethnicity (cOR 0.662, 95%CI 0.505–0.866), cardiovascular disease (cOR 1.357, 95%CI 1.115–1.652), immunocompromised (cOR 3.264, 95%CI 1.446–7.368), neurologic disease (cOR 1.630, 95%CI 1.283–2.072), diabetes (cOR 1.233, 95%CI 1.003–1.515), vaccination (cOR 0.694, 95%CI 0.516–0.933).

However, considering some important covariates such as sex could potentially contribute to the final odds of death, we also included the sex variable in the model regardless of *p*-value. In this way, there are total 10 covariates. To maintain the completeness, we include both models, which are model 1 (9 covariates), and model 2 (10 covariates, including sex), and report adjusted OR, Hosmer–Lemeshow test, ROC curve, VIF, AIC, c-statistic for both models. The sensitivity analysis was done on model 2.

**Fig. 2:**
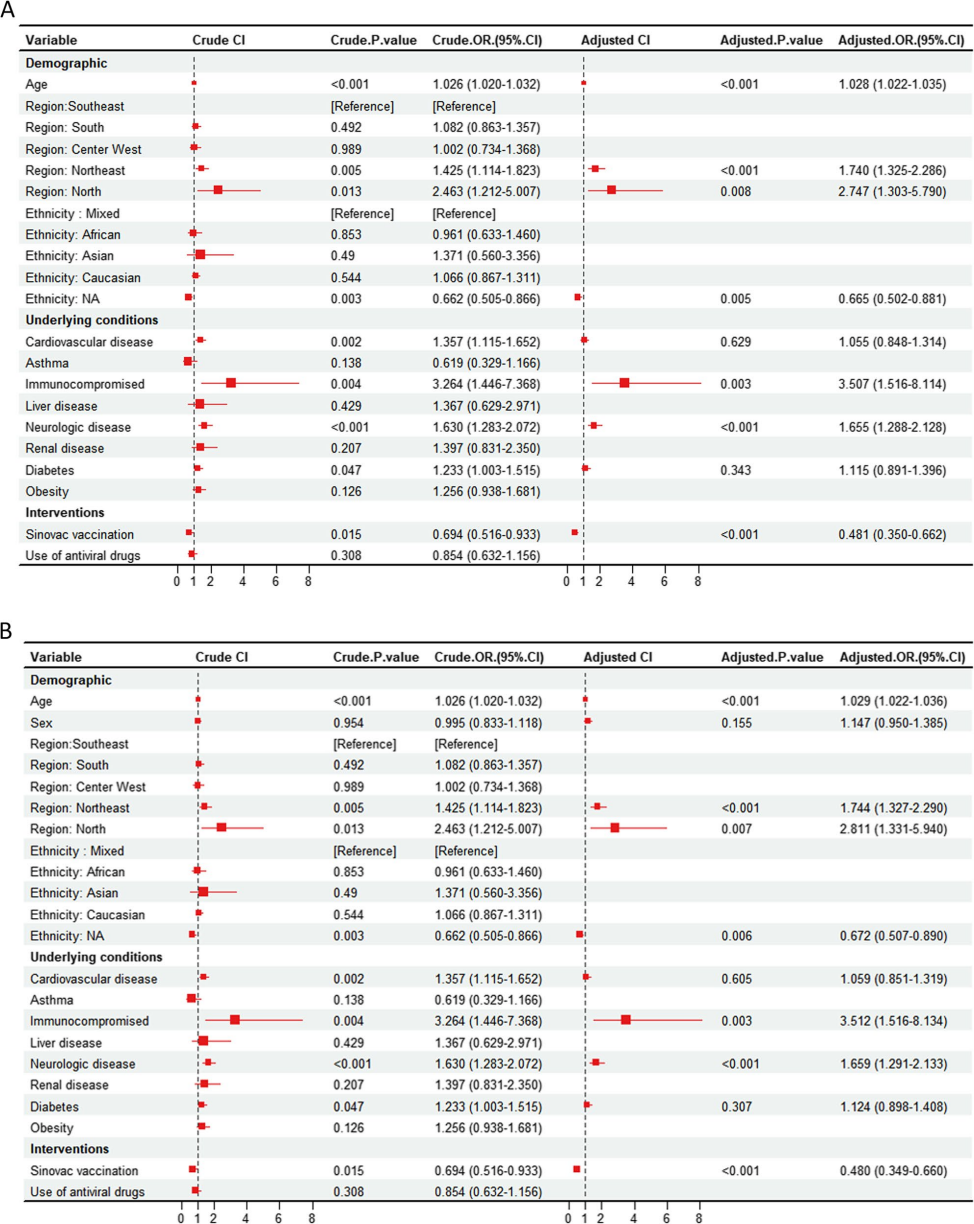
**A** Model 1, crude and adjusted odds ratio (cOR & aOR) for the variates associated with the risk of in-hospital mortality in schizophrenia patients with COVID-19, Brazil. The OR values were calculated using logistic regression. Squares represent odds ratios; error bars represent 95% confidence interval. The dashed vertical line represents the reference line, indicating an odds ratio equal to 1. The ethnicity indigenous is not included in this figure due to the excessive value (aOR: 1811639645.2, *p* > 0.9999). **B** Model 2, crude and adjusted odds ratio (cOR & aOR) for the variates associated with the risk of in-hospital mortality in schizophrenia patients with COVID-19, Brazil

For model 1, the adjusted OR values were listed below: age (adjusted OR [aOR] 1.028, 95%CI 1.022–1.035), northeast region (aOR 1.740, 95%CI 1.325–2.286), north region (aOR 2.747, 95%CI 1.303–5.790), NA ethnicity (aOR 0.665, 95%CI 0.502–0.881), cardiovascular disease (aOR 1.055, 95%CI 0.848–1.314), immunocompromised (aOR 3.507, 95%CI 1.516–8.114), neurologic disease (aOR 1.655, 95%CI 1.288–2.128), diabetes (aOR 1.115, 95%CI 0.891–1.396), sinovac vaccination (aOR 0.481, 95%CI 0.350–0.662). These data were summarized in Fig. 2A. For model 2, the adjusted OR values were also listed below: age (aOR 1.029, 95%CI 1.022–1.036), sex (aOR 1.147, 95%CI 0.950–1.385), northeast region (aOR 1.744, 95%CI 1.327–2.290), north region (aOR 2.811, 95%CI 1.331–5.940), NA ethnicity (aOR 0.672, 95%CI 0.507–0.890), cardiovascular disease (aOR 1.059, 95%CI 0.851–1.319), immunocompromised (aOR 3.512, 95%CI 1.516–8.134), neurologic disease (aOR 1.659, 95%CI 1.291–2.133), diabetes (aOR 1.124, 95%CI 0.898–1.408), Sinovac vaccination (aOR 0.480, 95%CI 0.349–0.660). These data were summarized in Fig. 2B.

**Fig. 3:**
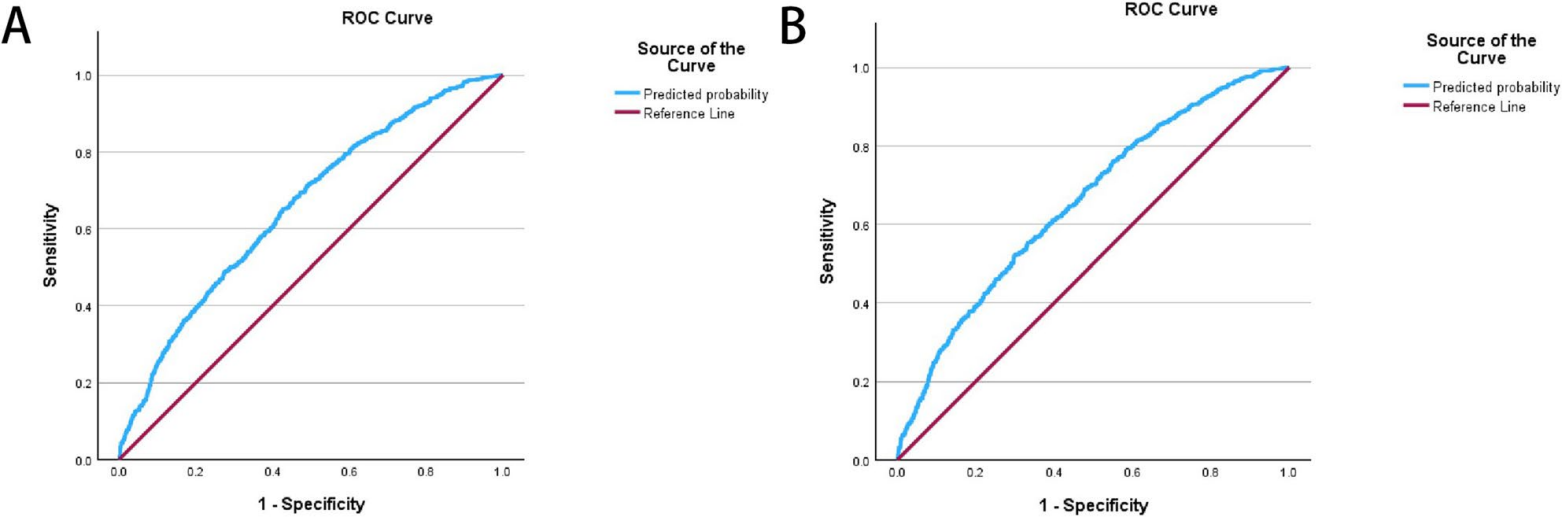
The ROC curve of model 1 (**A**) and model 2 (**B**). The blue curved line represents the model curve, the red line represents the reference line. AUC for model 1 is 0.657, AUC for model 2 is 0.657

The *p*-value of Hosmer–Lemeshow test for model 1 is 0.071, for model 2 is 0.420, which are both over 0.05, suggesting both models fit well. The AUC of model 1 is 0.657 (95%CI 0.633–0.681), and model 2 is 0.657 (95%CI 0.633–0.681). Both show fair discrimination (Fig. 3).

In model 1, the VIF values for covariate region, age, ethnicity, condition of chronic cardiovascular disease, condition of immunocompromised, condition of chronic neurologic disease, condition of diabetes, vaccinated with Sinovac are respectively 1.338, 1.129, 1.328, 1.148, 1.008, 1.017, 1.099, 1.079. In model 2, the VIF values for covariate region, age, sex, ethnicity, condition of chronic cardiovascular disease, condition of immunocompromised, condition of chronic neurologic disease, condition of diabetes, vaccinated with Sinovac are respectively 1.344, 1.148, 1.034, 1.337, 1.149, 1.008, 1.017, 1.102, 1.080. The VIF values of all covariates in both models are less than 10, showing there is no severe multicollinearity.

The AIC value for model 1 is 2604.526, for model 2 is 2604.494. The c-statistics for model 1 is 0.657, for model 2 is 0.657.

For sensitivity analysis, we kept using the same set of covariates as in the main analysis in model 2, focusing on southeast region subjects. A new logistic regression model was developed (model 3). The adjusted OR values for each covariate are listed below: age (aOR 1.025, 95%CI 1.016–1.034), sex (aOR 1.246, 95%CI 0.951–1.634),NA region (aOR 0.590, 95%CI 0.379–0.920), cardiovascular disease (aOR 1.118, 95%CI 0.823–1.519), immunocompromised (aOR 1.388, 95%CI 0.467–4.126), neurologic disease (aOR 1.806, 95%CI 1.279–2.551), diabetes (aOR 1.129, 95%CI 0.817–1.560), Sinovac vaccination (aOR 0.450, 95%CI 0.283–0.718). The p-value of Hosmer–Lemeshow test is 0.234. The AUC is 0.643 (95%CI 0.608–0.677). The VIF values are listed below: 1.138 (age), 1.033 (sex), 1.044 (ethnicity), 1.128 (cardiovascular disease), 1.009 (immunocompromised), 1.010 (neurologic disease), 1.088 (diabetes), 1.085 (Sinovac vaccination). The AIC is 1272.954. The c-statistics is 0.643.

## Discussion

### Main findings

We found that receiving Sinovac vaccine can significantly reduce the in-hospital mortality in individuals with SARS-CoV-2 infection and schizophrenia, compared to those not-vaccinated (aOR 0.480, 95%CI 0.349–0.660, *p* < 0.001). This suggests that the odds of death in the vaccinated group were 52% lower compared to that of the not-vaccinated group, which proved the effectiveness of Sinovac vaccine and its benefits on the prognosis on individuals with SARS-CoV-2 infection and schizophrenia. The AUC value of the ROC curve for our logistic regression model showed some power of discrimination.

### Implications of main findings

For a period, people were not very confirmed about the effectiveness of vaccine on schizophrenia patients. One study proposes that individuals with severe mental illness may develop a weaker immune response to vaccinations, potentially diminishing their effectiveness [20]. Our findings proved the effectiveness of Sinovac vaccine in reducing in-hospital mortality in schizophrenia patients infected with COVID-19, which is similar to a previous study in Israel [21]. The exact mechanisms for the effectiveness of Sinovac vaccine are not well understood. The possible explanation may be that the vaccine itself is potent enough to induce schizophrenia patients to produce abundant antibodies against COVID-19.

Since the onset of the COVID-19 pandemic, a growing number of research has demonstrated that individuals with mental health disorders face an elevated risk of severe COVID-19 complications. However, there is less vaccination rate in individuals with mental illnesses [22]. Another study found that individuals with schizophrenia experienced a significantly longer delay in receiving vaccination [21]. Our findings implicated that the government should pay more attention to schizophrenia patients, prompt timely vaccination among these patients, mitigating health inequalities to protect these disadvantaged groups.

### Implications of covariates

Based on different considerations, 2 models were established, which has been illustrated in the result section. In the discussion section, model 2 is used to analyze, because the covariates included by this model are more comprehensive.

In model 2, some covariates have fair predictive value in in-hospital mortality, including age, northeast region, north region, NA ethnicity, immunocompromised condition, neurologic disease. We found that older age can slightly increase the in-hospital mortality in individuals with SARS-CoV-2 infection and schizophrenia (aOR 1.029, 95%CI 1.022–1.036, *p* < 0.001), which is not surprising, because older people have less effective immune systems against COVID-19. This result is consistent with another retrospective observational study, which found that the average age of patients with schizophrenia who died of SARS-CoV-2 was notably higher compared to those who survived (57.71 ± 2.41 vs. 36.77 ± 9.55, *p* < 0.001) [23]. This suggests that health promotion policies need to be accurately stratified based on patients’ age, and the health of older groups needs priority attention.

We also found that patients living in northeast region (aOR 1.744, 95%CI 1.327–2.290, *p* < 0.001) and north region (aOR 2.811, 95%CI 1.331–5.940, *p* = 0.007) can increase the in-hospital mortality, compared to those living in southeast region. The potential explanation for this may be due to scarce medical resources in these remote and impoverished regions, compared to the southeast region. One report states that the north region of Brazil is the second poorest region after northeast region [24]. Data also shows that the market for private health plans and insurance is heavily concentrated in the southeast region [24]. These suggest that medical resources should be allocated more to these impoverished areas, and tax may be imposed on the south-east region, earmarked for the construction of health facilities in the north and northeast.

Being “NA ethnicity” can also reduce the in-hospital mortality (aOR 0.672, 95%CI 0.507–0.890, *p* = 0.006). compared to Mixed (Hispanic) ethnicity, which is the largest ethnic group in Brazil. This result may conflict with a previous systematic review, which summarized 11 studies, and found underprivileged racial and ethnic minority communities were more likely to be infected by SARS-CoV-2 compared to the majority groups in the region [25]. The explanation varies, because all other ethnicities may be included in this data missing (NA) group, making it hard to separate independent factors. However, this result still indicates that the government should be committed to eliminating racial inequalities in access to medical services.

For the underlying conditions, immunocompromised status (aOR 3.512, 95%CI 1.516–8.134, *p* = 0.003) and neurologic disease (aOR 1.659, 95%CI 1.291–2.133, *p* < 0.001) can increase the in-hospital mortality. This result is consistent with a previous review, suggesting that schizophrenia and COVID-19 share traits of energy metabolism dysregulation, immune disruption, and CNS abnormalities, while immune dysfunction may further worsen CNS abnormalities in both conditions, thus increasing mortality [26]. This suggests that patients with these underlying conditions should receive special attention.

### Implications of sensitivity analysis

In sensitivity analysis, we only focused people on the southeast region of Brazil, and similar results were obtained compared with the main analysis. The OR of Sinovac vaccination is 0.450, which is even better than that at the national level, which is 0.480. Besides, the cardiovascular disease, immunocompromised condition, and diabetes covariates are unsignificant statistically. There are various explanations. The southeast region has abundant medical resources, which provide easier access to hospital care, compared to other regions. People living there may have a better control over underlying conditions.

### Strength

Our study utilized the Brazilian SIVEP-Gripe database, which provides comprehensive, standardized, and systematically recorded data. This ensures reliability and a large sample size for analysis. We focused on schizophrenia patients, addressed a critical and often overlooked subgroup at high risk for COVID-19 outcomes. Moreover, this study used rigorous statistical methods to analyze. We employed robust multivariable logistic regression analysis to adjust for confounding and used AUC as well as VIF to confirm the goodness-of-fit and non-multicollinearity of the model.

### Limitation

However, the current study has some limitations that should be paid attention to. First, our study lacks data on antipsychotic medication use, which may affect immune response and clinical outcomes in schizophrenia patients. Vaccines can potentially affect drug metabolism, which may lead to reduced activity of certain cytochrome P450 enzymes. Consequently, this could cause significant changes in the serum levels of specific drugs following vaccination [27]. Second, this study does not distinguish between different COVID-19 variants, which may influence vaccine effectiveness and mortality rate. It is found that Omicron variant had much faster spreading rate than Delta variant, and can escape the neutralizing antibodies that were generated before [28]. Third, we only include data on Sinovac administration, but does not differentiate between the number of doses received, which may ignore the benefits of booster doses. Booster dose can reduce the transmission of COVID-19, lowering both symptomatic and asymptomatic infections [29]. Fourth, we created a “missing” category to mitigate the influence brought by missing data, which may introduce bias, because we are not sure whether the missingness is random. Fifth, for each comorbidity (e.g. immunocompromised), there is no standardized diagnosis method, because resources vary across the country. This may affect the quality of these data, but all diagnoses were carried out by qualified medical practitioners.

### Future research directions

Future research may focus on the effectiveness of COVID-19 vaccines on other severe mental illness patients, such as bipolar disorder and depression. We can compare the effectiveness of various COVID-19 vaccine platforms (e.g., mRNA, inactivated virus, protein subunit) in schizophrenia patients to identify the most effective approach. Additionally, we can investigate the reasons behind lower vaccination rates and delays among individuals with schizophrenia and design targeted interventions to promote timely vaccination.

## Conclusion

In summary, our study showed that receiving Sinovac vaccine can significantly reduce the in-hospital mortality in individuals with SARS-CoV-2 infection and schizophrenia, compared to those not-vaccinated. This proved the effectiveness of Sinovac vaccine in schizophrenia patients. Our findings suggest that the government should prioritize schizophrenia patients, encourage timely vaccination within this population, and address health disparities to safeguard these vulnerable groups.

## Data Availability

The datasets generated and/or analysed during the current study are available in the SIVEP-Gripe repository, https://opendatasus.saude.gov.br.
